# Identification of a novel pathogenic variant in *PALB2* and *BARD1* genes by a multigene sequencing panel in triple negative breast cancer in Morocco

**DOI:** 10.7150/jgen.61713

**Published:** 2021-09-18

**Authors:** Abdelilah Laraqui, Mathias Cavaillé, Nancy Uhrhammer, Oubaida ElBiad, Yannick Bidet, Hicham El Rhaffouli, Hicham El Anaz, Driss Moussaoui Rahali, Jaouad Kouach, Khaled Guelzim, Bouabid Badaoui, Abderrahman AlBouzidi, Mohammed Oukabli, Rachid Tanz, Yasser Sbitti, Mohammed Ichou, Khaled Ennibi, Yassine Sekhsokh, Yves-Jean Bignon

**Affiliations:** 1 Unité de séquençage, Centre de virologie, des maladies infectieuses et tropicales, Hôpital militaire d'Instruction Mohammed V, Faculté de Médecine et de Pharmacie, Université Mohammed V, Rabat, Maroc.; 2 Laboratoire de Recherche et de Biosécurité P3, Hôpital Militaire d'Instruction Mohammed V, Faculté de Médecine et de Pharmacie, Rabat, Maroc.; 3 Laboratoire Diagnostic Génétique et Moléculaire, Centre Jean Perrin, 58 rue Montalembert, Clermont-Ferrand, France.; 4 INSERM, U1240 Imagerie Moléculaire et Stratégies Théranostiques, Université Clermont Auvergne, Clermont-Ferrand, France.; 5 Laboratoire de Biodiversité, Ecologie et Génome, Faculté des Sciences, Université Mohammed V, Rabat, Maroc.; 6 Service de Gynécologie Obstétrique, Hôpital Militaire d'Instruction Mohammed V, Faculté de Médecine et de Pharmacie, Rabat, Maroc.; 7 Laboratoire d'Anatomopathologie, Hôpital Militaire d'Instruction Mohammed V, Faculté de Médecine et de Pharmacie, Rabat 10000, Maroc.; 8 Service d'Oncologie Médicale, Hôpital Militaire d'Instruction Mohammed V, Faculté de Médecine et de Pharmacie, Rabat, Maroc.

**Keywords:** Triple negative breast cancer, Next Generation Sequencing, Multigene panel testing, ACMG-AMP guidelines, Moroccan Population

## Abstract

Pathogenic variants (PVs) in *BRCA* genes have been mainly associated with an increasing risk of triple negative breast cancer (TNBC). The contribution of PVs in non-BRCA genes to TNBC seems likely since the processing of homologous recombination repair of double-strand DNA breaks involves several genes. Here, we investigate the susceptibility of genetic variation of the *BRCA* and non-*BRCA* genes in 30 early-onset Moroccan women with TNBC.

**Methods:** Targeted capture-based next generation sequencing (NGS) method was performed with a multigene panel testing (MGPT) for variant screening. Panel sequencing was performed with genes involved in hereditary predisposition to cancer and candidate genes whose involvement remains unclear using Illumina MiSeq platform. Interpretation was conducted by following the American College of Medical Genetics and Genomics-Association for Molecular Pathology (ACMG-AMP) criteria.

**Results:** PVs were identified in 20% (6/30) of patients with TNBC. Of these, 16.7% (5/30) carried a *BRCA* PV [10% (3/30) in *BRCA1*, 6.7% (2/30) in *BRCA2*] and 6.6% (2/30) carried a non-*BRCA* PV. The identified PVs in *BRCA* genes (*BRCA1* c.798_799delTT, *BRCA1* c.3279delC, *BRCA2* c.1310_1313del, and *BRCA2* c.1658T>G) have been reported before and were classified as pathogenic. The identified founder PVs *BRCA1* c.798_799del and *BRCA2* c.1310_1313delAAGA represented 10% (3/30). Our MGPT allowed identification of several sequence variations in most investigated genes, among which we found novel truncating variations in *PALB2* and *BARD1* genes. The *PALB2* c.3290dup and *BARD1* c.1333G>T variants are classified as pathogenic. We also identified 42 variants of unknown/uncertain significance (VUS) in 70% (21/30) of patients with TNBC, including 50% (21/42) missense variants. The highest VUS rate was observed in *ATM* (13%, 4/30). Additionally, 35.7% (15/42) variants initially well-known as benign, likely benign or conflicting interpretations of pathogenicity have been reclassified as VUS according to ACMG-AMP.

**Conclusions:** *PALB2* and *BARD1* along with *BRCA* genetic screening could be helpful for a larger proportion of early-onset TNBC in Morocco.

## Introduction

Breast cancer (BC) is the most commonly diagnosed malignancy and the leading cause of cancer death among women worldwide. An estimated 19.3 million new cases and 10 million cancer deaths occurred in 2020 1. According to the GLOBOCAN Cancer Tomorrow prediction tool, incident cases are expected to increase by more than 46% by 2040 2.The increasing global BC burden is mainly observed in low and medium human development index (HDI) countries 3, particularly women under the age of 50. Marked changes in lifestyle, socio-cultural contexts, and built environments are having a major impact on the prevalence of risk factors for BC burden in lowand medium HDI countries 4. In North Africa (i.e., in Morocco, Algeria, Tunisia, Libya and Mauritania) BC has rapidly overtaken cervical cancer as the most commonly diagnosed cancer 5. The incidence among women aged 15-49 is lower than in Western countries, but the very low incidence among women over 50, combined to the young age pyramid of North-Africa, makes the relative proportions of young patients substantially higher (50-60% versus 20% in France) 5. The size and grade of breast tumors are increased, while the median age of onset (48) is more than ten years younger than the European/North American median of 61 6. Moreover, the relative frequency of triple negative and inflammatory breast cancer is also higher in North Africa 5.

BC is a heterogeneous and polygenic disease that can be divided into different molecular sub-types based on histological and genomic features. Increasing steroid hormone receptors expression (estrogen and/or progesterone receptors) defines different BC subtypes using immunohistochemistry (IHC) markers together with clinic-pathologic indexes. Approximately 70% of BCs are estrogen receptors (ER) positive (ER+) and/or progesterone receptor (PR) positive (PR+) tumors 7. The human epidermal growth factor receptor type (HER) 2 amplification defines a second type, with an incidence of about 20% of BCs 8. The remaining belongs to the triple negative breast cancer (TNBC) subtype, i.e. those that do not over-express ER, PR or HER2. TNBC is associated with advanced-stage disease and higher-grade tumors at diagnosis and is associated with an increased recurrence risk and poor five-year survival rates relative to other BCs 9.

Current evidence suggests the association between specific molecular subtypes and *BRCA* mutational status. *BRCA1* PV carriers mainly increase TNBC, whereas *BRCA2* carriers are more likely to increase ER+ and/or PR+ tumors 10. Besides BRCA genes, an increasing number of studies have investigated genetic predisposition to TNBC using gene panel analysis 11,12. PVs in non-*BRCA* genes have been observed in women with TNBC, and subsequent studies showed that PVs in *BARD1, BRIP1, PALB2, RAD51C,* and *RAD51D* are more common in TNBC compared to other BC subtypes 13. PVs in established BC genes as well as other cancer susceptibility genes were identified in 14.4% (8.4% BRCA genes and 6.0% non-*BRCA*) of TNBC patients. PVs in *BARD1*, *BRCA1*, *BRCA2*, *PALB2*, and *RAD51D* were associated with high risk (Odds Ratio (OR)> 5.0) of TNBC. PVs in *BRIP1*, *RAD51C*, and *TP53* were associated with moderate risk (OR > 2) of TNBC 11. Moreover, PVs in *BARD1, RAD51C,* and *RAD51D* placed the patient at moderate risk for ER-BC and TNBC. Conversely, PVs in *ATM*, *CDH1*, and *CHEK2* were associated with ER+ BC. Additionally, there was a higher prevalence of PVs in *BRCA1*, *BRCA*/2 and *PALB2* observed with TNBC compared with ER+ BC, 8.13% versus 1.84%, respectively 14.

In North Africa, *BRCA* mutations frequency varied widely from ≈1% (Morocco) in sporadic BC 15 to 37.5% (Tunisia) in hereditary breast or/and ovarian cancer patients (HBOC) 16. The pooled prevalence of *BRCA*PVs among HBOC was 16% 17. However, the contribution of *BRCA* and non-*BRCA* genes to TNBC has not yet been determined. In a recent study from Morocco, it has been reported that 22% of TNBC patients harbor PVs in the *BRCA* genes using Ion AmpliSeq *BRCA1* and *BRCA2* Panel 18. Thus, there is a great need to investigate the frequency and importance of PVs in *BRCA* and non-*BRCA* genes among TNBC patients. Herein, we present the first Next Generation Sequencing (NGS)-based Multigene Panel Test (MGPT) study of 30 early onset (≤ 41 years of age) Moroccan women with TNBC. Panel sequencing was performed with genes involved in hereditary predisposition to cancer and candidate genes whose involvement remains unclear. This provides an advantage to map susceptibility genes for TNBC in North Africa.

## Methods

### Study subjects

Newly diagnosed women with TNBC were identified at the department of Obstetrics and Gynecology of Mohammed V Military Teaching Hospital in Rabat. TNBC patients were chosen according to the following criteria: age at diagnosis ≤ 41 years without family history or belonging to a family history. This family history of cancer was defined as diagnosed BC or OC in first- and second-degree relatives. Clinico-pathological data of TNBC, including tumor site, histological type and grade and TNM classification were collected at BC diagnosis. A total of 30 early onset TNBC patients were enrolled between January 2020 and June 2020, including 18 (60%) without a family history and 12 (40%) with a family history. Informed consent was obtained from all participants at the time of peripheral blood draw. Patients consenting to participate in the study completed epidemiology and family history questionnaires and donated 10 ml of blood for genetic analyses.

### Immunohistochemical analysis

The status of ER, PR, and HER2 receptors was determined using IHC analysis. Briefly, IHC analysis to determine ER and PR status was performed using standard procedures on 4 μm sections of paraffin embedded tissue specimens stained with the monoclonal antibodies 6F11 and 1A6 for ER and PR, respectively. Nuclear staining 1% was considered a positive result. The Hercept test was carried out in the institute since 2007 and was determined for all patients during the course of this study. Assays are scored with a 4-tiered system (0 - 3+). HER2 positivity was defined as strong complete membrane staining in at least 10% of tumor cells. Patients were considered HER2 if they had IHC 3+ by DAKO Hercept test. Tumors exhibiting equivocal HER2 expression, denoted as 2+ membranous staining of tumor cells, are confirmed by fluorescence *in situ* hybridization (FISH) at an outside laboratory. A signal ratios (HER2: CEP17) of ≥ 2.2 were classified as amplified. In the absence of positive FISH data, tumors scored 2+ by IHC were considered negative for HER2.

### DNA isolation

Genomic DNA was isolated from 200 μl peripheral blood anti-coagulated with Ethylene diamine tetra acetic acid on Blood DNA Maxi kit (Qiagen, Hilden, Germany) following the manufacturer's manual. DNA concentrations were assessed with the dsDNA HS assay kit by the Qubit 4.0 Fluorometer (Thermo Fisher Scientific, Waltham, USA).

### Gene panel testing

We performed targeted capture sequencing with a gene panel that is associated with high, intermediate and low cancer risk and candidate genes whose involvement remains unclear. Gene panel was designed by “Département d'Oncogénétique, Centre Jean Perrin, Clermont-Ferrand, France” according to the literature.The mode of inheritance is dominant in *AIP*, *APC*, *ATM*, *BAP1*, *BARD1*,*BMPR1A*, *BRCA1*, *BRCA2*, *BRIP1*, *CASR*, *CDC73*, *CDH1*, *CDK4*, *CDKN2A*, *CHEK2*, *EPCAM*, *FANCM*, *FH*, *FLCN*, *MAX*, *MCIR*, *MEN1*, *MET*, *MITF*, *MLH1*, *MSH2*, *MSH6*, *MUTYH*, *NBN*, *NF1*, *NF2*, *PALB2*, *PMS2*, *POLD1*, *POLE*, *PTEN*, *RAD50*, *RAD51C*, *RAD51D*, *RET*, *SDHA*, *SDHAF2*, *SDHB*, *SDHC*, *SDHD*, *SMAD4*, *SMARCA4*, *STK11*, *TMEM127*, *TP53*, and *VHL*. Additional genes such as *BRK1*, *FAM175*, *GREM1*, *MLH3*, *MRE11*, *MMSH2*, *NTHL1*, *PMS1*, *RAD51*, *RAD51B*, *RINTI*, *RNF3*, *RNF43* and *WRN* were also included in the testing panel as candidate genes. The genes with a known association with BC are *ATM*, *BARD1*, *BRCA1*, *BRCA2*, *CHEK2*, *MSH6*, *NF1*, *PALB2*, *PTEN*, *RAD51C*, *RAD51D*, and *TP53*. A list of analyzed genes is provided in Table 1.

### Panel sequencing

Sonic fragmentation of DNA was performed on a Bioruptor instrument (Diagenode). Kapa HTP library preparation and SeqCap EZ Choice probes and reagents (Roche) were used for library preparation and capture. Briefly, 20ng of genomic DNA was fragmented and processed by end-repairing, A-tailing and adapter ligation of paired-end indexed adapters, and a 7-cycle pre-capture PCR amplification. Further, the libraries were enriched through hybrid capture based method using specific probes. This was followed by PCR based enrichment, cleanup, and quantification of double stranded DNA using high sensitivity Qubit (Invitrogen, USA) measurement. Agencourt AMPure XP beads (Beckman Coulter, USA) were widely used for PCR amplicon purification and DNA size selection. The quality of fragmentation, library, and capture were evaluated using the Agilent 2100 Bioanalyzer system. A final library concentration ranging from 8 to 10 pM was used to carry out cluster generation and was sequenced on a Standard Flow Cell using V2 sequencing reagent kit (300 cycles) on MiSeq Instrument (Illumina, San Diego, CA). A typical sequencing run consists of 12 samples. All steps were performed following the providers' guidelines. No analysis of exons 11 to 15 of *PMS2* and exons 1, 13, and 14 of *SDHA*, and no quantitative analysis of *WRN* exon 10 were performed, due to high identity with paralogs genes.

### Bioinformatics Analysis

De-multiplexing was performed using bcl2fastq2 Conversion Software (Illumina). Sequencing reads were aligned to the University of California Santa Cruz hg19 reference genome assembly by Burrows-Wheeler Aligner 19. Recalibration of base quality scores (BaseRecalibrator) and realignment (RealignerTargetCreator, IndelRealigner) were carried out using Genome Analysis Toolkit (GATK) and PICARD tools, as recommended by Eurogentest guidelines 20. A variant calling was made using GATK HaplotypeCaller and annotated using Ensemble Variant Effect Predictor 21.Variants were filtered for quality score ≥ 30, depth ≥ 30x, and presence in ≥ 20% of reads.

### Interpretation of variants

An Interpretation of variants was conducted by following the classification system recommended by the American College of Medical Genetics and Genomics-Association for Molecular Pathology (ACMG-AMP) Standards and Guideline for the Interpretation of Sequence Variants 22. The process can result in 1 of 5 classifications: benign, likely benign, unknown/uncertain significance (VUS), likely pathogenic, and pathogenic. Likely benign and benign variants were not clinically reported. All classifications were ultimately evaluated by AL, MC and referred to NU. The clinical significance of each sequence variant was also based on a set of criteria such as allele frequency as well as the information from clinical genome databases including ClinVar (https://www.ncbi.nlm.nih.gov/clinvar/).

### *In silico* prediction

A potential clinical effect of VUS was evaluated by the analysis of the severity of the amino acid changes and their conservation across species. These analyses were performed using the Mutation Taster through Alamut® Visual v.2.11.0 including Alignment-Grantham variation Grantham deviation (Align GVGD; http://agvgd.iarc.fr/agvgd_input.php), Polymorphism Phenotyping-2 (Poly-Phen-2; http://genetics.bwh.harvard.edu/pph2/), and Sorting Intolerant from Tolerant (SIFT; http://blocks.fhcrc.org/sift/SIFT.html) scores. The Alamut interactive software provides results and/or links to the following databases used for variant annotation: Exome Aggregation Consortium (ExAC), Genome Aggregation Database (gnomAD: https://gnomad.broadinstitute.org/), Database of Short Genetic Variation (dbSNP) and ClinVar.

### Complementary analysis

Identified PVs were confirmed on a second patient sample. PVs in *BRCA*and* BRCA2* geneswere examined by NGS with Ion AmpliSeq *BRCA1* and *BRCA2* Panel (Life Technologies). The Sanger sequencing was performed for the novel PV in *PALB2* and *BARD1* genes using a 3500xl instrument and Big Dye terminator kit 3.1 (Applied Biosystems).

### Statistical analysis

The statistical analysis used the chi-squared test, with *p*< 0.05 taken as the threshold for a significant difference.

## Results

The mean age at diagnosis of TNBC was 38 (± 2.8) years. Most TNBC patients were diagnosed with infiltrating ductal carcinoma (IDC). The Scarff-Bloom-Richardson grades II and III were predominant (33.3% and 36.7% of cases, respectively). Axillary lymph nodes contained metastasis (N+) in 5 (16.7%) cases. Unilateral BC was diagnosed in all patients, and one (3.3%) had both BC and OC. The characteristics of the early onset TNBC patients are summarized in Table 2.

PVs were identified in 20% (6/30) of patients with TNBC. Of these, 16.7% (5/30) carried a *BRCA* PV [10% (3/30) in *BRCA1*, 6.7% (2/30) in *BRCA2*] and 6.6% (2/30) carried anon-*BRCA* PV. The identified PVs in *BRCA1* (*BRCA1* c.798_799delTT, *BRCA1* c.3279delC) and in *BRCA2* (*BRCA2* c.1310_1313del and *BRCA* 2 c.*1658T*>G) have been reported before and were classified as pathogenic (class 5). The identified founder *BRCA1* c.798_799del and *BRCA2* c.1310_1313delAAGA accounted for 10% (3/30) of all identified PVs. The *BRCA1* c.798_799del (p.Ser267LysfsX19) variant was found in two unrelated TNBC patients. Both carriers were diagnosed less than 38 years of age and showed a strong family history of BC. The *BRCA1* c.798_799delTT variant, located in exon 11, is a frame-shift variant including two small deletions, two bases (TT) deletion. The deletion causes a frame-shift which changes a Serine to a Lysine at codon 267, and creates a premature stop codon at position 19 of the new reading frame. The *BRCA1* c.798_799delTT variant, previously reported as *BRCA1* 917_918delTT using alternate nomenclature, has been reported in association with familial and early-onset BC and OC and has been described as a North African identified founder variant 15, 23-26.

The *BRCA1* c.3279delC (p.Tyr1094IlefsX15) variant was detected in a young woman diagnosed with TNBC at the age of 37. The *BRCA1* c.3279delC variant, located in exon 11, is a frame-shift variant including one small deletion, one base (C) deletion. The deletion causes a frame-shift which changes a Tyrosine to an Isoleucine at codon 1094, and creates a premature stop codon at position 15 of the new reading frame. Using alternate nomenclature, The *BRCA1* c.3279delC variant would be defined as *BRCA1*c.3390delC.

The *BRCA2* c.1310_1313delAAGA (p.Lys437Ilefs) variant, a specific founder variant from the North-East of Morocco 27, was detected in early onset women diagnosed with both TNBC (at the age of 35) and OC (at the age of 38). The *BRCA2* c.1310_1313delAAGA variant, located in exon 10, causes a frame-shift which changes a Lysine to an Isoleucine at codon 437 and creates apremature stop codon at position 22 of the new reading frame. It has previously reported as *BRCA2* 1310del4, *BRCA2*1537del4, and *BRCA2*1538del4 using alternate nomenclature.

The *BRCA2* c.1658T>G (p.Leu553Ter) variant, located in exon 11, causes a non-sense substitution. It was observed in one patient without family history, diagnosed with TNBC at the age of 36. In databases, the*BRCA2*c.1658T>G variant was associated with breast-ovarian cancer, hereditary cancer-predisposing syndrome, or hereditary breast or/and ovarian cancer syndrome.

Our panel sequencing allowed identification of several sequence variations in most investigated genes, among which we found novel variation in *PALB2* and *BARD1* genes. The *PALB2* c.3290dup (p.Lys1098) variant has not been reported before in the BIC database. The *PALB2* c.3290dup variant was detected in a young patient diagnosed with TNBC at an age ≤ 36 years. Family history was negative in the PV carrier. Thus, we concluded that this PV is not linked to family history of BC and/or OC. The *PALB2* c.3290dup variant is a frame-shift variation due to the insertion of C nucleotide at acid 3290 of codon 1097 in exon 12, which is predicted to lead to a premature stop codon 1098 and a truncated protein. The *BARD1* c.1333G>T (p.Glu445), that has not been reported previously, is classified as pathogenic (Class 5). The *BARD1* c.1333G>T variant occurred with *BRCA2* c.1658T>G (p.Leu553Ter) in early onset TNBC women diagnosed with pancreatic cancer at the age of 32. The early disease onset and having two cancers in this case may be the result of harboring two PVs in *BRCA2* and *BARD1* genes. Details of PVs detected by NGS-based MGPT in our study are reported in Table 3.

We also identified 42 VUS in 70% (21/30) of TNBC patients, including 50% (21/42) missense variants. The other VUSs were characterized as intronic or synonymous variants. A high rate was expected given the number of genes included in our panel gene. The BC susceptibility gene carrying the highest number of VUS was *ATM* (13%,4/30). Additionally, 35.7% (15/42) variants initially well-known as benign, likely benign or conflicting interpretations of pathogenicity have been reclassified as VUS according to the ACMG-AMP classification. Details of the identified VUS are shown in Table 4.

## Discussion

The absence of specific molecular markers for TNBC has made the targeted treatments extremely challenging and the death rates very high compared to the other BC subtypes. The NGS offers several clinical applications in cancer and precision oncology that are significant for risk predictors, early detection of disease, diagnosis by sequencing and medical imaging, accurate prognosis, biomarker identification and identification of therapeutic targets for novel drug discovery 28. To identify TNBC patients who might benefit from treatment strategies, *BRCA* and non-*BRCA* genes testing through NGS could lead to a more accurate prediction of the responsiveness to platinum and poly (ADP-ribose) polymerase (PARP) inhibitors. Thus, this strategy should be considered in management and precision medicine 29. Recent data have hypothesized that patients with advanced-stage TNBC associated with PVs in *BRCA* genes might be specifically sensitive to PARP inhibition; both Olaparib and Talazoparib are currently approved for such situation 30,31. Moreover, there is some evidence that adding platinum-agents in the neoadjuvant setting improves the pathologic complete response 32,33. The role of PARP inhibitors in the setting of non-*BRCA* associated cancers has been limited. Recently, Lapatinib plus Veliparib therapy have a manageable safety profile and promising antitumor activity in advanced TNBC 34.

### TNBC and *BRCA* genes

Genetic susceptibility to TNBC has been associated with rare germline variants occurring in *BRCA* genes, and *BRCA2* PVs were less common than *BRCA1* PVs 10,35. In our study, the *BRCA* PV prevalence was 16.7% among TNBC women aged <41. Among *BRCA* carriers, *BRCA1* gene was found to be mutated in 10% (3/30), while *BRCA2* gene was mutated in 6.7% (2/30). Our findings highlight that the TNBC phenotype at young age at onset can provide a valuable tool for identifying individuals with high likelihood of being *BRCA*PV carriers. This information emphasizes the recommendation for genetic testing in women diagnosed with TNBC at a young age because they have an increased risk of carrying *BRCA* PVs particularly in *BRCA1*. Data from a previous Tunisian study showed that diagnosis before the age of 40 could be the effective *BRCA* testing selection criterion among women with triple-negative tumors 36. Recent findings highlight that receptor triple negative could be an effective selection of patients for *BRCA1* analysis and should therefore be considered in genetic screening guidelines in Tunisia 37. In Algeria, the *BRCA1* PVs have been detected in Algerian patients with TNBC diagnosed at age ≤45 38. Thus, an earlier age should be considered as a guideline for *BRCA* genetic testing in women with TNBC in North Africa.

By analyzing the incidence rates of *BRCA1* PVs in both TNBC and non-TNBC, Tun et al. found that women with high-risk TNBC are much more likely to have PVs in *BRCA1* gene compared with women with non-TNBC and provides a relative risk of 5.65 (95% CI, 4.15-7.69). Furthermore, two out of nine (≈22%) TNBC patients harbor a PV in *BRCA1* gene 39. Armstrong et al. reported a concordant finding, although the estimates of *BRCA1* PV prevalence were mostly lower than the estimate by Tun et al. 40. In four studies of TNBC patients that reported on *BRCA* PVs prevalence, values ranged from 9.3% in an Australian study (n=439) 35 to 15.4% in a US study (n=207) 41. Both of these studies were done in early and advanced tumor stages BC populations. In UK, Robertson et al. showed that diagnosis of TNBC below 50 years would be a suitable age threshold for *BRCA* testing and may be a cost effectiveness strategy 42. Lu et al. recommended genetic testing for TNBC patients diagnosed before the age of 50, a population with *BRCA* PV frequency of 17.5%. The authors also highlighted data showing that the estimated *BRCA* PV frequency of individuals with any type of BC diagnosed before the age of 40 is 11% 43. Overall, testing in the UK has an unusually high threshold in comparison with the other European countries, where in all cases <41 years would be eligible for screening 44.

When stratified by family history, our study showed that 13% (4/30) early onset TNBC patients with a family history were identified with PVs in *BRCA* genes, compared to 3.3% (1/30) without a family history. Couch et al. revealed that 12.2% (66/539) TNBC patients with a family history carry *BRCA* PVs, compared to 8.6% (83/969) patients without a family history 12. Hartman et al. identified 21 *BRCA* PVs (13 in *BRCA1* and 8 in *BRCA2*) in a cohort of 199 unselected women with TNBC and providing an overall prevalence rate of 10.6%. Additionally, 5.2% (8/153) PVs were found in 153/199 patients without significant family history 45. In an unselected cohort study in 77 TNBC patients, it was found that 19.5% (15/77) had *BRCA* PVs including 15.6% (12/77) in *BRCA1* and 3.9% (3/77) in *BRCA* 46. In a Canadian TNBC cohort (n=54) with no familial BC aggregation, 9% (5/54) with PVs in *BRCA1* and 2% (1/54) of patients with PVS in *BRCA2* were detected 47. Lu et *al*. stated the importance of *BRCA* testing regardless of family history as few female family members or small families can mask genetics relative to BC and OC 43. Muendlein et al. conclude that the prevalence of *BRCA* PVs is high in TNBC patients and are not restricted to young women or patients with a positive family history 48. Although family history is commonly used to identify individuals with a possible predisposition to BC, Zang et al. showed that family history could not predict an underlying predisposition cancer syndrome in most patients. Furthermore, some individuals with cancer have de novo mutations, whereas others inherit them with incomplete penetrance; where, the family history is likely to be negative 49.

### TNBC and non *BRCA*-genes

In recent years it has become clear that truncating *PALB2* variants have been shown to be associated with a high risk for breast, ovarian and pancreatic cancers 50-52. In our investigation, the truncating variant *PALB2* c.3290dup was found in 3.3% (1/30) of TNBC cases. According to the ACMG-AMP guidelines, the *PALB2* c.3290dup variant was characterized as pathogenic (class 5) and considered predisposing for TNBC. Family history was negative in a *PALB2* c.3290dup carrier. Genetic testing for *PALB2* would provide another key genetic marker to identify women at elevated risk of TNBC regardless of their family history of BC. Our results suggest that the *PALB2* gene contributes to the risk of TNBC in North Africans and highlights the need to identify PVs in the *PALB2*gene which has a significant impact on an individual's risk of TNBC in younger women.

Similar observations are made when comparing the frequency of PVs reported here to that reported in the study of Zanati et al. in which 4.3% of TNBC patients carried PVs in *PALB2* 52. The observed frequency of *PALB2* PVs in our study (3.3%) and the last one appears to be higher compared to other populations (1.3-1.4%) 11,13. Low prevalence of *PALB2* germline mutation (~1%) was observed in 347 Australian TNBC women, similar to the prevalence of *PALB2* germline mutation of 1% in familial non-*BRCA* genes BC cohorts 11. *PALB2* PVs might be overrepresented in patients with TNBC in earlier studies performed in European cohorts 54,55. It is estimated that 30% 56 and 34% 57 of BC patients with a germline *PALB2* PV have a TNBC subtype. Shimelis et al. found that PVs in *PALB2* were found to be associated with a high-risk of TNBC with an OR of 14.41 (95%CI; 9.27-22.60) and were enriched in patients with TNBC compared to non-TNBC tumors with an OR of 2.12 (95%CI; 1.63-2.74) 11. The relatively poor survival from BC in patients with a *PALB2* PV was not attributable to the high prevalence of triple-negative phenotype 57. Due to the lower PV carrier frequency in the *PALB2* gene in the population, broad-based studies are needed to refine the genetic testing criteria and the management of the patients and their family members.

*BARD1* targeted sequencing studies showed that PVs in *BARD1* gene are enriched in TNBCs, which are associated with higher rates of recurrence, progression, and mortality 12,13. *BARD1* PVs were increased by more than threefold in TNBC cases (0.67%) compared to non-TNBC cases (0.18%), suggesting that *BARD1* is a predominantly TNBC predisposition gene 11. In our study, the *BARD1*c.1333G>T variant was identified in 3.3% (1/30) of TNBC women. According to the ACMG-AMP guidelines, *BARD1* c.1333G>T was characterized as pathogenic (class 5).

Similar observations are made when comparing thefrequency of *BRAD1*PVs reported here to that reported in previous studies. De Brakeleer et al. suggest that TNBC patients are enriched for PVs in *BRAD1* ascompared to control samples and high BCrisk families when they identified four harbored variants in *BARD1*, of which two protein-truncating variants (c.1347A>G and c.1972C>T) have been confirmed as pathogenic 58. In a study of 105 women with TNBC from a trial exploring the anti-tumor activity of neoadjuvant carboplatin/docetaxel chemotherapy, *BARD1* PVs were detected in two TNBC patients (1.9%) 59. The Analysis of 1824 TNBC patients unselected for age or family history of BC led to the identification of 0.5% (9/1824) cases with a *BARD1*-truncating variant 12. In Buys's study, the prevalence of *BARD1* PVs was higher among women with TNBC (3.3%) than among women with non-TNBC (1.7%) 13. Shimelis et al. identified 25 individuals harboring *BARD1* PVs (0.61%) and reported an OR of 5.92 for TNBC cases of African American and Caucasian populations 11. Rofes et al. identified ten *BARD1* PV carriers from 680 TNBC patients (carrier frequency = 0.9%), resulting in an OR = 5.40 60. Similar observations were reported in other studies 61,62, indicating that *BARD1* is a risk gene for TNBC. Although the *BARD1*gene offers a new hope for improving the TNBC therapy, the low number of *BARD1* PV carriers, the non-identification of a recurrent hotspot variant, a study with an sufficient sample size, a lack of geographically matched population controls have complicated the interpretation of the results and could hamper the strength of the association of *BARD1* PVs with TNBC risks 56. Further studies in larger cohorts will be necessary to more precisely assess the *BARD1*-associated risk with this tumor phenotype.

### TNBC and VUS

VUS represent a significant proportion of variants identified in clinical genetic testing, which account for about 40% of the total variants 63. In our study, 42 VUS were identified in 70% (21/30) of TNBC patients including 50% (21/42) distinct missense variants. The gene with the highest VUS frequency was the *ATM* (13%, 4/30). The *ATM* is a very large gene and is one of the genes with more identified VUS (40%) including missense, in-frame, or synonymous mutations 63. In our MGPT, the genes that contain more VUS are among the top ten genes cited in the literature with the highest number of variants submitted. A previous study has shown that the rate of VUSs was proportional to the number of genes analyzed in the MGPT in TNBC 64. Identification of the VUS has become a daily fact of life when tailoring genetic counseling, but little guidance is available for how best to approach them, and limited data are available on how they are affecting the medical practice and the well-being of cancer patients 65. In the lack of reliable clinical information or functional evidence the VUS remains non-informative in risk management and decision making.

Management recommendations for diagnostic and treatment decision-making for the carriers of PVs in BRCA and non *BRCA*-genes have been established. Recommendations are focused on a combination of annual magnetic resonance imaging (MRI) and mammography for women with familial risk or *BRCA* PV and a history of BC 66-68. Guidelines also made recommendations for the treatment of individuals with a *BRCA* carrier or those with a strong familial risk of developing BC. More recent European and US NCCN guidelines have updated recommendations regarding BRCA-targeted PARP inhibitor therapy in BC 69. Current evidence suggests that the BC risk for *PALB2* mutation carriers may overlap with that for *BRCA2* mutation carriers, particularly in the context of a significant family history 55. Accordingly, recently introduced NCCN practice guidelines suggest that a *PALB2* carrier should undergo a MRI or a mammography screening 70,71. Several studies have shown that the BARD1 can potentially become a new target for BC treatment. Li *et al.* have reported that the *BARD1* BRCT domain interacts with PAR, which results in a subsequent recruitment of the *BARD1-BRCA1* complex to the damaged DNA 72. A higher *BARD1* and *BRCA1* expression is associated with a worse prognosis of early BC patients, especially the ones that received a radiotherapy, indicating the potential use of PI3K inhibitors to reverse chemoresistance and radioresistance in ER^+^BC patients 73. Mammography and MRI remain the fundamental imaging modalities for the high andvery high-risk BC patients. An interesting approach might be radiogenomics, which brings together the clinical assessment, imaging results, and the genetic background 74. This approach would be of interest in relation to the immunohistochemical staining of the *BARD1* gene, which in turn can be imaged inmagnetic resonance scans 75.

## Limitations

Our study has some limitations that should be acknowledged. The sample size was relatively small. Thus, our data underscore the need for larger series to better understand the frequency and the contribution of PVs in *BRCA, PALB2* and *BARD1* genes in patients with early onset TNBC. Despite the reduced sample size in our cohort, our findings supports those deleterious PVs in *PALB2* and *BARD1*were enriched in TNBC patients. Our analysis does not include copy number variants. Although having many advantages, large sequencing panels still have limitations compared to the traditional Sanger sequencing test or smaller NGS panels in cancer precision. In our MGPT, Some genes or exons are not well captured and therefore are not covered, resulting in some variants within these regions going undetected and hence being refractory to analysis. Despite these limitations, our findings may help in implementing effective strategies for *BRCA, PALB2* and *BARD1* PVs testing in TNBC patients.

## Conclusion

Defects in homologous recombination DNA repair genes that may be targeted with PARP inhibitors occur in 60 to 69% of TNBC. PARP repairs the damaged DNA and renders the tumor highly sensitive to platinum-based chemotherapy. With the rise of NGS, it is possible to target multiple genes rapidly and simultaneously in a cost-effective manner. Defining groups of TNBC patients with *BRCA, PALB2* and *BARD1* PVs is important for the clinical management of patients because several new treatment strategies are being evaluated for related tumors. Overall, an improvement in the detection rate by using an extensive panel analysis determines the hereditary cancer to which the patients and families are exposed based on their history and genetics as part of a targeted therapy 76.

## Supplementary Material

Supplementary figure s1.

Supplementary table S2.

Supplementary table S3.

## Figures and Tables

**Table 1:** Triple negative breast cancer susceptibility genes

Gene	Genomic location	Coding transcript	Tumor types	Cancer syndrome
*AIP*	11q13.3	NM_003977.2	Pituitary adenoma	pituitary syndrome
*APC*	5q22.2	NM_000038.5	colorectal, pancreatic, desmoid, hepatoblastoma, glioma, other CNS	adenomatous polyposis coli; Turcot syndrome
*ATM*	11q22.3	NM_000051.3	leukaemia, lymphoma, medulloblastoma, glioma	ataxia-telangiectasia
*BAP1*	3p21.1	NM_004656.2	mesothelioma, uveal melanoma	tumor predisposition syndrome
*BARD1*	2q35	NM_000465.2	ovarian cancer, breast cancer, endometrioid cancer	ovarian cancer, breast cancer, endometrioid cancer
*BMPR1A*	10q23.2	NM_004329.2	gastrointestinal polyps	juvenile polyposis
*BRCA1*	17q21.31	NM_007294.3	breast, ovarian	hereditary breast/ovarian cancer
*BRCA2*	13q13.1	NM_000059.3	breast, ovarian, pancreatic, leukaemia	hereditary breast/ovarian cancer
*BRK1*	3p25.3	NM_018462	renal	Von Hippel-Lindau (VHL) syndrome
*BRIP1*	17q23.2	NM_032043.2	AML, leukaemia, breast	Fanconi anaemia J, breast cancer susceptiblity
*CASR*	3q21.1	NM_000388.3	parathyroid, colorectal	hyperparathyroidism-jaw
*CDC73*	1q31.2	NM_024529.4	Parathyroid adenoma, multiple ossifying jaw fibroma	hyperparathyroidism-jaw tumour syndrome
*CDH1*	16q22.1	NM_004360.3	gastric	familial gastric carcinoma
*CDK4*	12q14.1	NM_000075.3	melanoma	familial malignant melanoma
*CDKN2A*	9p21.3	NM_000077.4	melanoma, pancreatic	familial malignant melanoma
*CHEK2*	22q12.1	NM_007194.3	breast	Familial breast cancer
*EPCAM*	2p21	NM_002354.2	colorectal	colorectal cancer, hereditary non polyposis, type 8
*FANCM*	14q21.3	NM_020937.2	breast, ovarian	hereditary breast/ovarian cancer
*FAM175*	4q21.23		breast, ovarian	hereditary breast/ovarian cancer
*FH*	1q43	NM_000143.3	leiomyomatosis, renal	hereditary leiomyomatosis and renal cell cancer
*FLCN*	17p11.2	NM_144997.5	renal, fibrofolliculomas, trichodiscomas	Birt-Hogg-Dube syndrome
*GREM1*	15q13.3	NM_013372	colorectal	hereditary mixed polyposis syndrome
*MAX*	14q23.3	NM_002382.4	pheochromocytoma	pheochromocytoma, susceptibility to
*MCIR*	16q24.3	NM_002386.3	skin	familial malignant melanoma
*MEN1*	11q13.1	NM_000244.3	Thyroid adenoma, pituitary adenoma, pancreatic islet cell, carcinoid	multiple endocrine neoplasia type 1
*MET*	7q31	NM_001127500.1	lung, gastric, colon	lung, gastric, and colon cancer
*MITF*	3p13	NM_000248.3	melanoma	melanoma, cutaneous malignant, susceptibility to, 8
*MLH1*	3p22.2	NM_000249.3	colorectal, endometrial, ovarian, central nervous system	hereditary non-polyposis colorectal cancer, Turcot syndrome
*MLH3*	14q24.3	NM_001040108.1	colorectal, endometrial	colorectal cancer, hereditary nonpolyposis, type 7, Endometrial Cancer
*MRE11*	11q21	NM_005591.4	colorectal	colorectal cancer
*MSH2*	2p21-p16	NM_000251.2	colorectal, endometrial, ovarian	hereditary non-polyposis colorectal cancer
*MSH6*	2p16.3	NM_000179.2	colorectal, endometrial, ovarian	hereditary non-polyposis colorectal cancer
*MUTYH*	1p34.1	NM_001128425.1	Colorectal	adenomatous polyposis coli
*NTHL1*	14q24.3	NM_001040108.1	colorectal, breast	colorectal cancer, breast cancer, and colorectal polyposis.
*NBN*	8q21.3	NM_002485.4	non-Hodgkin lymphoma, glioma, medulloblastoma, rhabdomyosarcoma	nijmegen breakage syndrome
*NF1*	17q11.2	NM_000267.3	neurofibroma, glioma	neurofibromatosis type 1
*NF2*	22q12.2	NM_000268.3	meningioma, acoustic neuroma	neurofibromin 2 (merlin)
*PALB2*	16p12.2	NM_024675.3	Wilms tumour, medulloblastoma, AML, breast	Fanconi anaemia N, breast cancer susceptibility
*PMS1*	2q31-q33	NM_000534.4	colorectal	hereditary nonpolyposis colorectal cancer type 3
*PMS2*	7p22.1	NM_000535.5	Colorectal, endometrial, ovarian, medulloblastoma, glioma	hereditary non-polyposis colorectal cancer, Turcot syndrome
*POLD1*	19q13.33	NM_001256849.1	colorectal	lynch syndrome
*POLE*	12q24.3	NM_006231.2	colorectal	colorectal cancer, adenomatous colorectal polyps, family histories of colorectal cancer
*PTEN*	10q23.31	NM_000314.4	Harmartoma, glioma, prostate, endometrial	Cowden syndrome, Bannayan-Riley-Ruvalcaba syndrome
*RAD50*	5q31.1	NM_005732.3	breast cancer	Nijmegen breakage syndrome-like disorder
*RAD51*	15q15.1	NM_002875.5	lung, colon, breast cancer	lung adenocarcinoma, glioblastoma, colon adenocarcinoma, breast invasive ductal
*RAD51B*	14q23-q24.2	NM_002877.5	lung, skin, bladder, endometrial , prostate	lung adenocarcinoma, cutaneous melanoma, bladder urothelial carcinoma, endometrial endometrioid adenocarcinoma, prostate adenocarcinoma
*RAD51C*	17q22	NM_058216.1	breast, ovarian cancer	breast-ovarian cancer, familial, susceptibility to, 3
*RAD51D*	17q12	NM_002878.3	breast, ovarian cancer	breast-ovarian cancer, familial, susceptibility to, 4
*RET*	10q11.21	NM_020975.4	medullary thyroid, papillary thyroid, pheochromocytoma	multiple endocrine neoplasia 2A/2B
*RINT1*	7q22.3	NM_021930.6	breast cancer, colon	breast cancer, Lynch syndrome
*RNF43*	17q23.2	NM_017763.4	colon, breast, pancreatic, endometrial, lung	colon adenocarcinoma, breast cancer , pancreatic adenocarcinoma, endometrial, lung adenocarcinoma
*SDHA*	5p15	NM_004168.2	lung, colon, breast, pancreatic, bladder	lung adenocarcinoma, colon adenocarcinoma, breast cancer, pancreatic adenocarcinoma, bladder urothelial carcinoma
*SDHAF2*	11q12.2	NM_017841.2	paraganglioma	familial paraganglioma
*SDHB*	1p36.13	NM_003000.2	paraganglioma, pheochromocytoma	familial paraganglioma
*SDHC*	1q23.3	NM_003000.2	paraganglioma, pheochromocytoma	familial paraganglioma
*SDHD*	11q23.1	NM_003002.2	paraganglioma, pheochromocytoma	familial paraganglioma
*SMAD4*	18q21.2	NM_005359.5	gastrointestinal polyp	juvenile polyposis
*SMARCA4*	19p13.3	NM_001128844.1	lung, colon, endometrial, bladder, breast	lung adenocarcinoma, colon adenocarcinoma, endometrial endometrioid adenocarcinoma, bladder urothelial carcinoma, breast invasive ductal carcinoma
*STK11*	19p13.3	NM_000455.4	Jejunal hamartoma, ovarian, testicular, pancreatic	Peutz-Jeghers syndrome
*TMEM127*	2q11.2	NM_017849.3	pheochromocytoma, renal cell carcinoma	pheochromocytoma, susceptibility to
*TP53*	17p13.1	NM_000546.5	breast, sarcoma, adrenocortical carcinoma, glioma, multiple other tumour types	Li-Fraumeni syndrome
*VHL*	3p25.3	NM_000551.3	renal, haemangioma, pheochromocytoma	Von Hippel-Lindau syndrome
*WRN*	8p12	NM_000553.4	colon, gastric	peritoneal cancer, colon cancer, and stomach cancer

**Table 2:** Demographic and clinical-pathologic characteristics of 30 TNBC samples

Characteristics	Patients
Age	38 (± 2.8)
**Family history**	
Positive	18 (60%)
Negative	12 (40%)
**Histologic**	
IDC	19 (63.3%)
Lolular	7 (23.3%)
Medullar	2 (6.7%)
Others	2 (6.7%)
**Tumour size**	
T1	5 (16.7%)
T2	10 (33.3%)
T3	11 (36.7%)
T4	4 (13.3%)
**SBR grading**	
I	7 (23.3%)
II	10 (33.3%)
III	11 (36.7%)
IV	2 (6.7%)
**Lymph node status**	
Positive	5 (16.7%)
negative	7 (23.3%)

IDC: invasive ductal carcinoma; SBR: Scarff-Bloom-Richardson.

**Table 3:** Pathogenic variants and likely pathogenic variant detected by NGS based-MGPT in Moroccan TNBC patients

	Age at diagnosis	Affected gene	Nucleotide change	Amino acid change	Type of variant	Rs number	ClinVar Database	ACMG Classification
1907L0132	38	*BRCA1*	c.798_799del	p.Ser267fs	Frameshift	rs80357724	Pathogenic	**5**
1907L0131	36	*BRCA1*	c.798_799del	p.Ser267fs	Frameshift	rs80357724	Pathogenic	**5**
1907L0133	40	*BRCA1*	c.3279del	p.Tyr1094Ilefs	Frameshift	rs397509050	Pathogenic	**5**
1907L0141	38	*BRCA2*	c.1310_1313del	p.Lys437Ilefs	Frameshift	rs80359277	Pathogenic	**5**
1907L0145	37	*BRCA2*	c.1658T>G	p.Leu553Ter	Nonsens	rs876659627	Pathogenic	**5**
		*BARD1*	c.1333G>T	p.Glu445	Missense	Not reported	Not reported	**5**
1907L0146	40	*PALB2*	c.3290dup	p.Lys1098	Frameshift	Not reported	Not reported	**5**

ACMG: American College of Medical Genetics and Genomics.

**Table 4:** Variant of unknown/uncertain significance detected by NGS based-MGPT in Moroccan TNBC patients

Gene	Sequence variant	Amino acid hange	Type of variant	Rs number	ClinVar Database	ACMG Classification
*APC*	c.-151G>C	Non coding	5 Prime UTR	rs1029997545	Likely benign​	3
*APC*	c.325C>T	p.Arg109Trp	Missense	Not reported	Not reported	3
*APC*	c.295C>T	p.Arg99Trp	Missense	rs139196838	Conflicting interpretations of pathogenicity	3
*APC*	c.835-41A>G	Non coding	Intron Variant	Not reported	Not reported	3
*APC*	c.781-41A>G	Non coding	Intron Variant	Not reported	Not reported	3
*ATM*	c.1595G>A	p.Cys532Tyr	Missense	rs35963548	Conflicting interpretations of pathogenicity	3
*ATM*	c.1810C>T	p.Pro604Ser	Missense	rs2227922	Conflicting interpretations of pathogenicity	3
*ATM*	c.9002G>A	p.Ser3001Asn	Missense	rs587781413	VUS	3
*ATM*	c.8560C>T	p.Arg2854Cys	Missense	rs201958469	VUS	3
*BARD1*	c.1028C>T	p.Thr343Ile	Missense	rs201032007	Conflicting interpretations of pathogenicity	3
*BRCA1*	c.3587C>T	p.Thr1196Ile	Missense	rs80356944	VUS	3
*BRIP1*	c.415T>G	p.Ser139Ala	Missense	rs202072866	VUS	3
*BMPR1A*	c.431-30A>G	Non coding	Intron Variant	Not reported	VUS	3
*CDKN2A*	c.13T>A	p.Phe5Ile	Intron Variant	rs776987532	VUS	3
*CDKN2A*	c.369T>A	p.His123Gln	Missense	rs6413463	Conflicting interpretations of pathogenicity	3
*FANCM*	c.1667A>G	p.Asp556Gly	Missense	rs148810507	VUS	3
*FANCM*	c.1576C>G	p.Leu526Val	Missense	rs144215747	VUS	3
*MET*	c.841T>G	p.Phe281Val	Missense	Not reported	Not reported	3
*MITF*	c.-28C>T	Non coding	5 Prime UTR	Not reported	Not reported	3
*MLH3*	c.3746C>T	p.Ser1249Phe	Missense	rs139265757	Benign​	3
*MSH2*	c.965G>T	p.Gly322Val	Missense	rs4987188	VUS	3
*MSH6*	c.2540A>T	p.Glu847Val	Missense	Not reported	Not reported	3
*NBN*	c.425A>G	p.Asn142Ser	Missense	rs769414	Conflicting interpretations of pathogenicity	3
*NF1*	c.8161-45A>C	Non coding	Intron Variant	rs17879551	Likely benign	3
*NF1*	c.8098-45A>C	Non coding	Intron Variant	rs17879551	Likely benign	3
*NTHL1*	c.86C>T	p.Pro29Leu	Missense	Not reported	Not reported	3
*PMSI*	c.-99G>T	Non coding	5 prime UTR	rs577363454	VUS	3
*PMS2*	c.250A>C	p.Thr84Pro	Missense	rs1554304938	VUS	3
*POLD1*	c.883G>A	p.Val295Met	Missense	rs199545019	Conflicting interpretations of pathogenicity	3
*POLD1*	c.2388+5G>A	Non coding	Intron Variant	rs750085275	VUS	3
*POLD1*	c.1014C>G	p.Cys338Trp	Missense	Not reported	Not reported	3
*RAD50*	c.2354C>T	p.Ala785Val	Missense	Not reported	Not reported	3
*RAD51B*	c.1050C>G	p.Cys350Trp	Missense	Not reported	Not reported	3
*RET*	c.1756C>T	Leu586Phe	Missense	rs777604634	VUS	3
*RNF43*	c.172A>G	Thr58Ala	Missense	Not reported	VUS	3
*RNF43*	c.2054C>A	Thr685Asn	Missense	Not reported	Not reported	3
*RNF43*	c.-611C>T	Non coding	Missense	rs62636625	Not reported	3
*SDHA*	c.1367C>T	Ser456Leu	Missense	rs76896145	Benign	3
*SMARCA4*	c.722_733del	Gly241_Pro244del	Inframe Deletion	rs568390760	Conflicting interpretations of pathogenicity	3
*SMARCA4*	c.-72C>T	Non coding	5 prime UTR	rs559144002	Likely benign	3
*WRN*	c.1530_1532del	Glu510del	Initiator Codon	rs781777438	VUS	3
*WRN*	c.3785C>G	Thr1262Arg	Missense	rs78488552	Conflicting interpretations of pathogenicity	3

ACMG: American College of Medical Genetics and Genomics; VUS: variant of uncertain significance.
