# Supplementary material for: Identification of a novel pathogenic variant in PALB2 and BARD1 genes by a multigene sequencing panel in triple negative breast cancer in Morocco
Source: J Genomics. 2021 Sep 18;9:43–54. doi: 10.7150/jgen.61713 (PMC8490085; doi:10.7150/jgen.61713)
Supplement: Supplementary file 1 — Supplementary figure s1. [file jgenv09p0043s1.pdf]

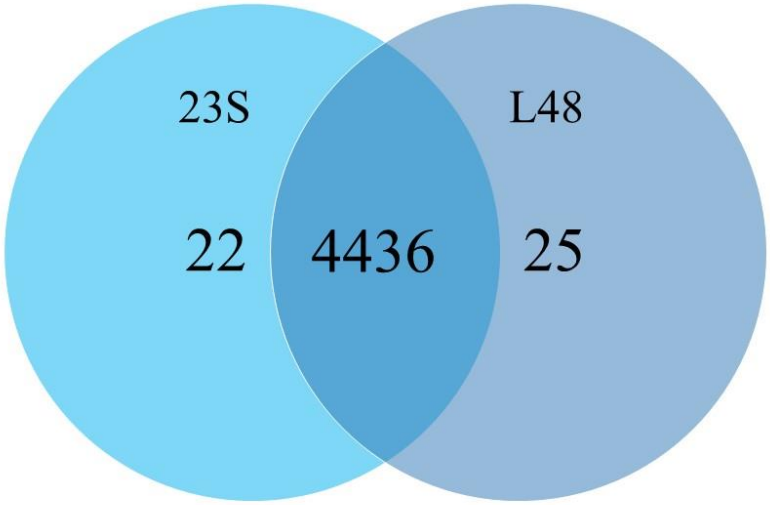

Supplementary Figure S1: Genome comparison between *Pseudomonas entomophila* 23S and L48 for orthologous genes.
